# Heavy Metal Concentrations and Health Risk Assessment of Melon Seeds (*Citrullus lanatus*) From Selected Markets in Ghana

**DOI:** 10.1155/ijfo/8819979

**Published:** 2026-09-21

**Authors:** Afia Sakyiwaa Amponsah, Gladys Kyere, Alex Amerh Agbeshie, Berlinda Agyei-Poku

**Affiliations:** ^1^ Faculty of Applied Science and Technology, Sunyani Technical University, Sunyani, Bono Region, Ghana, stu.edu.gh; ^2^ Department of Food and Nutrition Education, University of Education, Winneba, Central Region, Ghana, uew.edu.gh; ^3^ Department of Environmental Management, University of Energy and Natural Resources, Sunyani, Bono Region, Ghana, uenr.edu.gh

**Keywords:** dietary exposure, food safety, Ghana, hazard quotient, incremental lifetime cancer risk, melon seeds, oilseeds, toxic elements

## Abstract

Melon seeds (*Citrullus lanatus* (Thunb.) Matsum. & Nakai), locally known as ‘agushi’ in Ghana, are widely consumed as thickeners in soups and stews and are a valued oilseed crop, yet data on toxic element contamination from the Bono Region remain scarce. This study determined cadmium (Cd), arsenic (As), lead (Pb) and mercury (Hg) concentrations in melon seeds from six market locations across the Bono, Bono East and Ahafo Regions of Ghana, and evaluated health risks for adult males (body weight [BW] = 70 kg), adult females (BW = 60 kg) and children aged 6–12 years (BW = 26 kg); the sex‐differentiated assessment reflects the well‐established influence of body weight on mass‐normalised dietary exposure. Samples were oven‐dried, ground and acid‐digested using a concentrated HNO3/HClO4 (4:1, v/v) mixture on a programmable hot plate, then analysed by atomic absorption spectrophotometry. Mean concentrations ranged from 0.0017–0.0086 mg/kg (Cd), 0.0013–0.0027 mg/kg (As), 0.00103–0.00108 mg/kg (Pb) and 0.000130–0.000383 mg/kg (Hg), all substantially below EU 2023/915 maximum levels. Hazard indices (HI: 0.0091–0.0202, calculated using the USEPA IRIS 2025 updated RfD for As of 6.0 × 10^−5^ mg/kg/day) were well below 1.0, confirming negligible noncarcinogenic risk. Incremental lifetime cancer risk (ILCR) values (1.04 × 10^−6^–2.81 × 10^−6^) fell within the acceptable range (10^−6^–10^−4^). Melon seeds from the sampled markets are safe for regular consumption under current exposure conditions.

## 1. Introduction

Melon seeds (*Citrullus lanatus* (Thunb.) Matsum. & Nakai), locally referred to as ‘agushi’ or ‘akatoa’ in Ghana, are seeds from a cucurbitaceous plant belonging to the family Cucurbitaceae [1]. They are a key ingredient in Ghanaian traditional cuisine, widely used as thickeners in soups and stews, and are consumed as roasted snacks [2]. The Bono Region of Ghana is a major commercial hub for this commodity, with the Techiman market serving as one of the principal trading centres for melon seeds sourced from the region and neighbouring countries including Togo and Nigeria [3].

Heavy metals and metalloids, collectively termed toxic elements, belong to a category of environmental contaminants of significant food safety concern because of their persistence, nonbiodegradability and tendency to bioaccumulate in food chains [4]. These four elements (Cd, As, Pb and Hg) are among the most toxicologically relevant toxic elements in food systems. Cadmium (Cd) causes renal tubular dysfunction and nephropathy and is classified as a Group 1 carcinogen by the IARC [5]. Arsenic (As), strictly classified as a metalloid rather than a heavy metal, is a potent carcinogen in its inorganic form associated with cancers of the bladder, lung and skin [6]. Lead (Pb) impairs neurodevelopment in children and exerts haematotoxic and nephrotoxic effects in adults, with no established threshold for developmental toxicity [7]. Mercury (Hg), particularly in its inorganic form, targets the renal and central nervous systems [4].

Oilseeds, including melon seeds, can accumulate heavy metals and metalloids through soil‐to‐plant transfer, foliar deposition, irrigation water, post‐harvest contamination and storage [8]. In Ghana and West Africa, agricultural intensification, artisanal and small‐scale gold mining (ASGM) and the increased use of agrochemicals have raised concerns about potential contamination of locally produced foodstuffs [9]. Despite the growing interest in the nutritional characterisation of indigenous Ghanaian seeds [10], systematic studies on toxic element burdens specifically in melon seeds from the Bono Region are lacking.

The European Commission, [11], establishes legally enforceable maximum levels (MLs) for Cd, Pb and Hg in a wide range of foods including oilseeds; no specific ML for As in oilseeds is currently set under this regulation. These benchmarks are widely adopted as reference standards in sub‐Saharan African food safety assessments given the absence of nationally codified maximum limits in many jurisdictions including Ghana [12].

Quantitative health risk assessment provides a structured framework for translating measured food contaminant concentrations into consumer risk estimates, enabling differentiated evaluation across population subgroups with distinct body weights and dietary patterns [13]. Children are given specific attention because their lower body weight relative to food intake results in higher mass‐normalised exposures; they have biological systems and organs at various stages of development and they are more susceptible to several toxic element toxicants particularly Pb and Cd at given exposure levels [14].

This study therefore is aimed at (i) determining the concentrations of Cd, As, Pb and Hg in melon seeds from six market locations across the Bono, Bono East and Ahafo Regions of Ghana; (ii) comparing detected levels against EU 2023/915 [11] MLs; and (iii) conducting sex‐ and age‐differentiated health risk assessments including estimated daily intake (EDI), hazard quotient (HQ), hazard index (HI) and incremental lifetime cancer risk (ILCR) for adult males, adult females and children aged 6–12 years, using Ghana‐specific anthropometric reference values.

## 2. Materials and Methods

### 2.1. Study Area and Sample Collection

Melon seed samples were collected from six commercial market locations in the Bono, Bono East and Ahafo Regions of Ghana: Bechem, Berekum, Nkwanta, Nsawkaw, Sunyani and Techiman. These markets were purposively selected to represent the geographic spread of melon seed trading points within the region, spanning the regional capital (Sunyani) and major satellite trading centres. At each location, approximately 50 g of melon seeds were purchased from each of 10 independent vendors and combined to form a single pooled composite sample of approximately 500 g per location [15]. Three analytical replicates were prepared from each composite.

### 2.2. Sample Preparation and Acid Digestion

Samples were sorted to remove foreign matter, rinsed with deionised water and oven‐dried at 60°C to constant weight following AOAC Official Method 925.10 [16]. Dried samples were ground to a fine homogeneous powder using a stainless‐steel laboratory mill and stored in sealed polypropylene bags at 4°C until analysis. Acid digestion was performed following the procedure described by De Oliveira et al. [17] with minor modifications: approximately 0.5 g of each powdered sample was weighed into a Teflon vessel and digested with a mixture of concentrated HNO3 and HClO4 (4:1, v/v) on a programmable hot plate with temperature increments from 80°C to 180°C until a clear, near‐colourless solution was obtained. Digest solutions were filtered through Whatman No. 1 filter paper, made up to 50 mL with deionised water and stored at 4°C. Reagent blanks and certified reference materials (National Institute of Standards and Technology, SRM 1515 Apple Leaves) were digested concurrently for quality control.

### 2.3. Instrumental Analysis

Concentrations of Cd and Pb were quantified by flame atomic absorption spectrophotometry (FAAS), whereas As and Hg were determined by hydride generation AAS (HGAAS) to achieve the required detection sensitivity [17]. All measurements were made using a calibrated Perkin‐Elmer AAnalyst 400 AAS instrument at the KNUST Soil Science Laboratory. Standard calibration curves (R^2^ > 0.999) were prepared from certified standard solutions (1000 mg/L, Merck). The detection limits were 0.0001 mg/kg (Cd), 0.0002 mg/kg (As), 0.0001 mg/kg (Pb) and 0.00005 mg/kg (Hg).

### 2.4. Health Risk Assessment

The quantitative health risk assessment followed the framework of the United States Environmental Protection Agency [18]. Three population subgroups were assessed: adult males (BW = 70 kg), adult females (BW = 60 kg) and children aged 6–12 years (BW = 26 kg) [19] [20]. The body weight values of 70 kg for adult males and 60 kg for adult females were selected as population‐specific reference values appropriate for Ghanaian adults, consistent with anthropometric data reported in the Ghanaian literature [19, 20]. These values are also consistent with the EFSA Scientific Committee [21] guidelines for population‐level exposure assessment and with WHO/IPCS [22] risk assessment protocols. A standardised daily intake (DI) of 25 g/day was applied for both adult groups, based on reported melon seed consumption in Ghanaian markets [3]. Children′s DI was set at 10 g/day, derived by applying a standard body‐weight‐adjusted scaling factor to the adult DI: using the ratio of child to adult body weight (26 kg/60 kg ≈ 0.43) scaled against the adult DI of 25 g/day yields an estimated child DI of approximately 10–11 g/day [23]. This approach is consistent with standard paediatric scaling methods widely applied in dietary risk assessment [23].

#### 2.4.1. EDI

EDI (mg/kg BW/day) was calculated for each metal, location and population group from Equation 1 as follows:

(1) EDI=C×DIBW,

where C is the mean metal concentration in the seed (mg/kg), DI is the daily melon seed intake (kg/day) and BW is the mean body weight (kg) for each subgroup.

#### 2.4.2. Noncarcinogenic Risk (HQ and HI)

The HQ for each metal in Equation 2 was calculated as follows:

(2) HQ=EDIRfD,

where RfD is the oral reference dose (mg/kg/day) from the USEPA Integrated Risk Information System (IRIS). Values applied were: Cd = 1 × 10^−3^ mg/kg/day [24–27], As = 6.0 × 10^−5^ mg/kg/day (USEPA IRIS updated January 2025 value), and Hg (inorganic) = 3 × 10^−4^ mg/kg/day. In line with current USEPA IRIS and EFSA guidance, no oral RfD is applied for Pb; contemporary toxicological frameworks no longer support a threshold‐based RfD for Pb given the absence of a safe exposure level for its neurodevelopmental effects [26]. The HI was calculated as the sum of HQs for Cd, As and Hg. HQ or HI < 1.0 indicates negligible noncarcinogenic risk.

#### 2.4.3. ILCR

ILCR was estimated as in Equation 3:

(3) ILCR=EDI×CSF.

### 2.5. Statistical Analysis

Data are presented as means ± standard deviation (SD) of three analytical replicates (*n* = 3). Prior to inferential analyses, the assumptions of normality and homogeneity of variances were assessed using the Shapiro–Wilk test [28] and Levene′s test [29], respectively; both assumptions were satisfied for all datasets (*p* > 0.05). One‐way ANOVA followed by Tukey′s honestly significant difference (HSD) post hoc test was applied to compare toxic element concentrations across locations. Pearson′s correlation coefficients were calculated to examine interelement associations [30]. All analyses were performed in IBM SPSS Statistics Version 28.0. Statistical significance was accepted at *p* < 0.05.

## 3. Results and Discussion

### 3.1. Heavy Metal Concentrations and Regulatory Compliance

The mean concentrations of heavy metals and metalloid (±SD) in melon seeds from the six Bono Region market locations are presented in Table 1, alongside the applicable EU 2023/915 MLs. All detected mean concentrations were substantially below the respective EU regulatory thresholds, confirming broad regulatory compliance of Bono Region melon seeds.

**Table 1:** Mean concentrations of heavy metals and metalloid (mg/kg, wet weight, *n* = 3) in melon seeds from six Bono Region market locations and comparison with EU 2023/915 maximum levels.

Location	Cd (mg/kg)	As (mg/kg)	Pb (mg/kg)	Hg (mg/kg)	*N*
Bechem	0.0086 ± 0.0002^d^	0.0023 ± 0.0001^b^	0.00106 ± 0.000006^a^	0.000347 ± 0.000023^c^	3
Berekum	0.0035 ± 0.0001^c^	0.0017 ± 0.0002^a^	0.00105 ± 0.000035^a^	0.000250 ± 0.000040^b^	3
Nkwanta	0.0025 ± 0.0003^b^	0.0013 ± 0.0003^a^	0.00108 ± 0.000036^a^	0.000383 ± 0.000032^c^	3
Nsawkaw	0.0017 ± 0.0003^a^	0.0017 ± 0.0004^a^	0.00103 ± 0.000025^a^	0.000153 ± 0.000021^a^	3
Sunyani	0.0034 ± 0.0003^c^	0.0027 ± 0.0001^b^	0.00104 ± 0.000021^a^	0.000190 ± 0.000010^a^	3
Techiman	0.0018 ± 0.0001^a^	0.0019 ± 0.0003^a^	0.00104 ± 0.000010^a^	0.000130 ± 0.000010^a^	3
EU ML (2023/915)	0.50	—	0.10	0.05	—

*Note:* EU ML = maximum level per Commission Regulation (EU) 2023/915 for oilseeds: Cd 0.50 mg/kg, Pb 0.10 mg/kg, Hg 0.05 mg/kg; = no EU ML established for As in oilseeds. Values are mean ± SD (*n* = 3); different superscript letters within a column indicate significant differences (Tukey HSD *p* < 0.05).

^a^One‐way ANOVA: Cd F (5, 12) = 361.18,

^b^As F (5, 12) = 11.10,

^c^Hg F (5, 12) = 51.37 (all *p* < 0.001);

^d^Pb F (5, 12) = 1.55, *p* = 0.248 (ns).

#### 3.1.1. Cd

Cd concentrations ranged from 0.0017 ± 0.0003 mg/kg (Nsawkaw) to 0.0086 ± 0.0002 mg/kg (Bechem). All means were below 1.8% of the EU ML of 0.50 mg/kg for oilseeds (Annex I Section 3.2, EU 2023/915). These values are consistent with Cd levels reported in egusi melon powder from Ghanaian markets by Arthur et al. [15] and are lower than values recorded for melon seeds in Nigerian [31], suggesting a relatively low Cd burden in Bono Region seeds.

#### 3.1.2. As

As concentrations were uniformly low across all six locations, ranging from 0.0013 ± 0.0003 mg/kg (Nkwanta) to 0.0027 ± 0.0001 mg/kg (Sunyani). Commission Regulation (EU) 2023/915 does not establish a specific ML for As in oilseeds; accordingly, no direct EU ML comparison was applied for this metal. The low As burden is consistent with the limited arseniferous geology of the Bono Region and with As values reported for locally grown Cucurbitaceae seeds in West Africa [31]. As concentrations differed significantly across locations (F (5, 12) = 11.10, *p* < 0.001), with Sunyani recording the highest mean (0.0027 mg/kg) and Nkwanta the lowest (0.0013 mg/kg). Despite this statistical significance, all values were within a narrow absolute range and well below any established health‐based benchmark, suggesting that the differences, although real, may reflect minor variations in soil mineralogy or seed provenance rather than localised anthropogenic inputs.

#### 3.1.3. Pb

Pb concentrations had the lowest variability across all metals and all locations, ranging from 0.00103 ± 0.000025 mg/kg (Nsawkaw) to 0.00108 ± 0.000036 mg/kg (Nkwanta). Coefficients of variation within locations were below 5% for all sites, a pattern indicative of a common background signal rather than differential site‐level contamination. All Pb values were below 1.1% of the EU ML of 0.10 mg/kg. This result is markedly lower than Pb levels reported in egusi powder from Ghanaian markets by Arthur et al. [15], who detected values of 0.4–0.8 mg/kg and reported HQ > 1.0 for Pb, a contrast likely attributable to the use of raw whole seeds in the present study versus milled powder subject to postprocessing contamination. The low Pb values are broadly consistent with the progressive reduction in environmental Pb burden that followed the phase‐out of leaded petrol in Ghana and across West Africa [9].

#### 3.1.4. Hg

Hg concentrations were the lowest detected among all four metals, ranging from 0.000130 ± 0.0000100 mg/kg (Techiman) to 0.000383 ± 0.000032 mg/kg (Nkwanta), all below 0.77% of the EU ML of 0.05 mg/kg. These levels are consistent with the expected low Hg background in agricultural produce from areas without proximate Hg‐emitting industrial or artisanal mining activities. The northward proximity of the Bono Region to ASGM areas in Bono East and Ahafo Regions, where Hg amalgamation is practised, warrants periodic monitoring of both soil and seed Hg burdens in forthcoming surveys [32, 33].

### 3.2. EDI

EDIs for each metal, population group and location are presented in Table 2. In all cases, EDI values were several orders of magnitude below the respective USEPA oral reference doses, providing a preliminary indication of negligible exposure risk.

**Table 2:** Estimated daily intake (EDI × 10^−6^ mg/kg BW/day) of heavy metals and metalloid from melon seed consumption across all population groups.

Location	Adult male				Adult female				Children (6–12 years)			
	Cd	As	Pb	Hg	Cd	As	Pb	Hg	Cd	As	Pb	Hg
Bechem	3.08	0.821	0.380	0.124	3.60	0.958	0.443	0.145	3.32	0.885	0.409	0.133
Berekum	1.25	0.607	0.376	0.089	1.46	0.708	0.439	0.104	1.35	0.654	0.405	0.096
Nkwanta	0.89	0.464	0.386	0.137	1.04	0.542	0.450	0.160	0.96	0.500	0.415	0.147
Nsawkaw	0.62	0.618	0.369	0.055	0.72	0.721	0.430	0.064	0.67	0.665	0.397	0.059
Sunyani	1.21	0.954	0.370	0.068	1.42	1.113	0.432	0.079	1.31	1.027	0.399	0.073
Techiman	0.65	0.679	0.371	0.046	0.76	0.792	0.433	0.054	0.70	0.731	0.400	0.050
RfD (×10 − 3)	1.000	0.060	N/A	0.300	1.000	0.060	N/A	0.300	1.000	0.060	N/A	0.300

*Note:* All EDI values ×10^−6^ mg/kg BW/day. RfD row in ×10^−3^ mg/kg/day for comparative scale; As RfD = 6.0 × 10^−5^ mg/kg/day (updated USEPA IRIS 2025) expressed as 0.060 × 10^−3^; Pb row = N/A (no oral RfD per current USEPA IRIS/EFSA).

Abbreviations: BW, body weight; DI, daily intake.

As expected from the EDI formula, children displayed the highest EDI per kg of body weight due to their lower BW relative to the DI considered. However, even children′s EDI values for Cd at Bechem, the site with the highest Cd mean, reached only 3.32 × 10^−6^ mg/kg/day, compared with the Cd RfD of 1.00 × 10^−3^ mg/kg/day, a margin exceeding two orders of magnitude.

### 3.3. Noncarcinogenic Risk Assessment (HQ and HI)

Individual HQ values for Cd, As and Hg were well below 1.0 across all groups and sites (maximum HQ = 0.0186 for As at Sunyani, adult females, using the updated USEPA IRIS 2025 RfD of 6.0 × 10^−5^ mg/kg/day). Hazard indices reflecting cumulative exposure to Cd, As and Hg (Pb excluded) ranged from 0.0091 (adult males, Nkwanta) to 0.0202 (adult females, Sunyani), all more than 49‐fold below the regulatory threshold of 1.0, confirming no noncarcinogenic risk to any assessed population group from melon seed consumption at current market levels. The consistent HI < 1 pattern across all locations and demographic groups is in agreement with comparable assessments of traditional food commodities in West Africa; for instance, HQ and HI values below 1.0 have similarly been reported for market vegetables in Imo State, Nigeria [34] and for Jamaican food crops where cumulative HI remained below 0.34 across all sampled commodities [35].

Adult females recorded the highest HI at most locations owing to their lower body weight relative to adult males at the same DI. This inverse relationship between body weight and HI is a well‐recognised feature of USEPA‐based dietary risk assessments, where lower body weight yields a proportionally higher mass‐normalised EDI, and consequently a higher HQ for equivalent intake. Children′s HI values were intermediate between those of adult males and adult females at most locations, reflecting their lower DI (10 g/day vs. 25 g/day for adults) partially offsetting their lower body weight (26 kg vs. 60–70 kg); this pattern demonstrates that neither the DI nor the BW parameter alone drives risk classification, but rather their ratio. The observation that children′s HI values, despite their lower absolute DI (10 g/day vs 25 g/day for adults), remained comparable to those of adults further illustrates the dominant role of body weight normalisation in determining group‐specific risk; this finding is consistent with patterns reported in multigroup food safety assessments across sub‐Saharan Africa [36].

Across all groups, Cd and As contributed the largest proportions to the HI, consistent with their substantially lower oral reference doses (Cd: 1.00 × 10^−3^ mg/kg/day; As: 6.00 × 10^−5^ mg/kg/day [updated USEPA IRIS 2025]) compared with Hg (3.00 × 10^−4^ mg/kg/day); Pb was excluded from HI calculations per current toxicological guidance (see Section 2.4.2). The disproportionate contribution of Cd and As to the HI, even at relatively low absolute concentrations, reflects a pattern observed broadly in dietary risk assessments: in a study of fruits, vegetables and root crops in Jamaica, Cd accounted for approximately 63% of the total HI and As for a further 23%, even though both metals occurred below international maximum limits [35]. The dominance of Cd in the HI profile at Bechem, the site with the highest Cd concentration contrasts with the more As dominant HI pattern at other locations where Cd concentrations were lower, suggesting that the relative speciation of the HI is sensitive to site‐specific metal loading rather than reflecting a fixed elemental hierarchy. Nonetheless, no HI value approached the hazard threshold of 1.0 for any group or location, and the magnitudes observed here (all ≤ 0.01) are at least an order of magnitude lower than the HI values of 6.51–29.30 recorded for market vegetables in the Tamale Metropolis, Ghana, where Cd, Cr and Mn individually exceeded HQ = 1 in some food types [37].

These findings are more favourable than those reported by Arthur et al. [15] for Pb in processed egusi powder from Ghanaian markets, where Pb HQ values exceeded 1.0 and Pb concentrations reached 0.4–0.8 mg/kg, substantially higher than the levels observed here for raw seeds across the Bono Region. This contrast reinforces the importance of distinguishing between raw seed matrices and processed forms in risk characterisation. Postharvest processing operations, particularly milling using metallic attrition equipment widely employed in Ghanaian informal markets, are a documented route of Pb introduction into food powders, as mechanical wear from steel grinding surfaces can shed particulate metal directly into the product [15, 38]. Additional contamination pathways during processing include contact with metal storage containers and cross‐contamination during open‐market handling [38]. The present study, by focusing on raw seeds sampled prior to any milling or processing, therefore characterises the intrinsic, agriculturally derived contamination burden of the commodity and is not directly comparable to studies conducted on processed egusi products. This distinction has practical regulatory implications: risk management interventions for melon seeds in the Bono Region should prioritise monitoring of postharvest handling and processing infrastructure rather than restricting agricultural production. The HQ and HI values for all population groups and locations are summarised in Table 3.

**Table 3:** Hazard quotient (HQ) per metal and hazard index (HI) across all population groups. Adult male: BW = 70 kg, DI = 25 g/day; adult female: BW = 60 kg, DI = 25 g/day; children (6–12 year): BW = 26 kg, DI = 10 g/day.

Location	Adult male					Adult female					Children (6–12 years)				
	Cd	As	Pb	Hg	HI	Cd	As	Pb	Hg	HI	Cd	As	Pb	Hg	HI
Bechem	0.00308	0.01370	0.00011	0.00041	**0.01719**	0.00360	0.01595	0.00013	0.00048	**0.02005**	0.00332	0.01475	0.00012	0.00044	**0.01851**
Berekum	0.00125	0.01010	0.00011	0.00030	**0.01165**	0.00146	0.01180	0.00013	0.00035	**0.01361**	0.00135	0.01090	0.00012	0.00032	**0.01257**
Nkwanta	0.00089	0.00775	0.00011	0.00046	**0.00910**	0.00104	0.00905	0.00013	0.00053	**0.01062**	0.00096	0.00835	0.00012	0.00049	**0.00980**
Nsawkaw	0.00062	0.01030	0.00011	0.00018	**0.01110**	0.00072	0.01200	0.00012	0.00021	**0.01293**	0.00067	0.01110	0.00011	0.00020	**0.01197**
Sunyani	0.00121	0.01590	0.00011	0.00023	**0.01734**	0.00142	0.01855	0.00012	0.00026	**0.02023**	0.00131	0.01710	0.00011	0.00024	**0.01865**
Techiman	0.00065	0.01130	0.00011	0.00015	**0.01210**	0.00076	0.01320	0.00012	0.00018	**0.01414**	0.00070	0.01220	0.00011	0.00017	**0.01307**

*Note:* HQ = EDI/RfD; HI = sum of HQs for Cd, As and Hg (bold); Pb excluded per current USEPA IRIS/EFSA guidance (no oral RfD established for Pb). Values < 1.0 indicate negligible noncarcinogenic risk. RfD values (USEPA IRIS): Cd = 1 × 10^−3^, As = 6.0 × 10^−5^ (updated January 2025) and Hg = 3 × 10^−4^ mg/kg/day.

### 3.4. ILCR Assessment

ILCRs for Cd and As (Pb excluded per current USEPA IRIS and EFSA guidance in Section 2.4.3), along with cumulative ILCRs, are presented in Table 4. As and Cd were the dominant contributors to total ILCR across all groups and sites, a pattern directly attributable to their substantially higher oral cancer slope factors (As CSF: 1.50 (mg/kg/day)^−1^; Cd CSF: 0.38 (mg/kg/day)^−1^) as specified by USEPA IRIS [39–41]. Both As and Cd are classified as Group 1 human carcinogens by the International Agency for Research on Cancer. As is primarily associated with cancers of the skin, lung, bladder and liver, whereas Cd is linked to lung and renal carcinogenesis. The marked difference in slope factors means that even when absolute Cd and As concentrations are low relative to regulatory limits, they disproportionately dominate the carcinogenic risk profile in the ILCR framework; this effect has been documented in a range of dietary risk assessments, including studies of Jamaican food crops where Cd drove the majority of total ILCR despite concentrations well below international limits [42]; and in Nigerian drinking water where Cd accounted for the dominant share of cumulative ILCR across multiple water source types [43].

**Table 4:** Incremental lifetime cancer risk (ILCR × 10^−6^) from heavy metals and metalloid exposure via melon seed consumption across all population groups.

Location	Adult male				Adult female				Children (6–12 years)			
	Cd	As	Pb	Total	Cd	As	Pb	Total	Cd	As	Pb	Total
Bechem	1.171	1.232	0.003	**2.403**	1.363	1.438	0.004	**2.805**	1.261	1.327	0.003	**2.588**
Berekum	0.475	0.911	0.003	**1.386**	0.554	1.063	0.004	**1.617**	0.512	0.981	0.003	**1.493**
Nkwanta	0.339	0.696	0.003	**1.035**	0.396	0.813	0.004	**1.209**	0.365	0.750	0.004	**1.115**
Nsawkaw	0.235	0.927	0.003	**1.162**	0.274	1.081	0.004	**1.355**	0.253	0.997	0.003	**1.250**
Sunyani	0.461	1.430	0.003	**1.891**	0.538	1.669	0.004	**2.207**	0.497	1.540	0.003	**2.037**
Techiman	0.248	1.018	0.003	**1.266**	0.290	1.188	0.004	**1.478**	0.267	1.096	0.003	**1.363**

*Note:* All values ×10^−6^. ILCR = EDI × CSF. CSF values (USEPA IRIS): Cd = 0.38 (mg/kg/day)^−1^; As = 1.5 (mg/kg/day)^−1^ [27]. Pb and Hg excluded (no established oral CSF per current USEPA IRIS/EFSA guidance). Total = bold (Cd + As only). Acceptable range = 1 × 10^−6^–1 × 10^−4^ (USEPA, 2000).

Total ILCRs ranged from 1.04 × 10^−6^ (adult males, Nkwanta) to 2.81 × 10^−6^ (adult females, Bechem). The USEPA has proposed an acceptable or tolerable cancer risk range of 10^−6^–10^−4^, indicating that a risk between one‐in‐a‐million and one‐in‐ten‐thousand is considered acceptable; risks below 1 × 10^−6^ are deemed negligible, whereas risks exceeding 1 × 10^−4^ are considered unacceptable. In the present study, ILCR fell within this acceptable range and were concentrated in the lower portion of that range between approximately 10 and 28 times the lower boundary of 10^−6^, reflecting a risk profile closer to the negligible end of the spectrum than to the threshold of regulatory concern. This is consistent with the outcomes of comparable dietary ILCR assessments for food commodities sampled from uncontaminated or low‐anthropogenic‐impact areas across Africa and beyond, where total ILCRs within the 10^−6^ to 10^−5^ range are widely reported for traditionally consumed plant foods [42].

Adult females recorded the highest ILCRs at most sites because their lower body weight (60 kg vs. 70 kg for males) produces a higher mass‐normalised EDI at the same DI. This inverse relationship between body weight and ILCR is a systematic feature of the USEPA chronic DI framework, not a site‐specific finding and is consistently reported across multigroup ILCR assessments in the literature [21]. The Nkwanta site returned the lowest total ILCRs across all groups (1.04–1.21 × 10^−6^), which are at the lower boundary of the USEPA acceptable range and approach the threshold below which risk is classified as negligible (≤1 × 10^−6^), whereas the Becham site returned the highest (2.41–2.81 × 10^−6^), driven by its higher Cd concentration, yet remained more than 35‐fold below the upper threshold of 1 × 10^−4^.

Pb was excluded from carcinogenic risk calculations because current USEPA IRIS and EFSA frameworks no longer support a linear oral cancer slope factor for Pb; the evidence base does not permit reliable establishment of a carcinogenic threshold for dietary Pb exposure. This exclusion is consistent with contemporary risk assessment practice [44]; the noncarcinogenic neurotoxic and developmental effects of Pb at low exposure levels are independently well documented and are captured through the EDI‐based assessment framework where appropriate [39]. It should also be noted that the As‐derived ILCR values in this study are calculated from total As concentrations. Since inorganic As, rather than organic forms such as arsenobetaine, is the fraction responsible for carcinogenicity [44], the true carcinogenic risk attributable to As from melon seed consumption may be somewhat lower than the modelled values, as a proportion of the measured total As will be present in less bioavailable and noncarcinogenic organic forms. Future speciation analyses would be needed to refine this estimate.

### 3.5. Comparative Context and Limitations

The metal concentrations and associated health risk estimates obtained in this study compare favourably with published data for melon seeds and related Cucurbitaceae seeds in West Africa. Ohiagu et al. [31] reported Cd values of 0.01–0.06 mg/kg in *Cucumeropsis mannii* seeds from Nigeria, higher than the values found here. The current Cd concentration, (0.0017–0.0086 mg/kg) were lower than the 0.015–0.021 mg/kg reported in processed egusi powder from Ghanaian markets by Arthur et al. [15] suggesting that milling and processing operations may contribute to Cd accumulation beyond background seed levels. Arthur et al. [15] reported Pb concentrations of 0.40–0.80 mg/kg in egusi powder sold in Accra markets. Arthur et al. [15] themselves reported HQ > 1 for Pb under their own exposure assumptions, in sharp contrast to the very low Pb concentrations observed here in raw seeds. This marked difference reinforces the view that metallic attrition from milling equipment, rather than agronomic or soil‐derived contamination, is the principal driver of Pb risk in melon seed products in Ghana These comparative findings suggest that Bono Region melon seeds are among the less contaminated in the West African context, potentially reflecting lower use of heavy‐metal‐containing agrochemicals in the sampling areas.

Limitations of this study include the cross‐sectional nature of sampling (single time point), which precludes assessment of seasonal variation; the use of simplified point estimates for consumption (DI) rather than probabilistic distributions; the application of population‐level BW values rather than individual‐level data; and the absence of data on background heavy metal exposures from other dietary sources, which would be needed for cumulative dietary exposure assessment. Additionally, the DI estimates used for children are conservative approximations; children in West African households may consume varying proportions of melon seed‐containing dishes depending on meal composition and portion size. Future studies should incorporate dietary recall data to improve DI estimation precision for each subgroup.

## 4. Conclusion

This study provides a systematic characterisation of heavy metal contamination and differentiated consumer health risks for melon seeds (*C. lanatus*) from the Bono, Bono East and Ahafo Region of Ghana, encompassing adult males, adult females and children. Concentrations of Cd, Pb and Hg were well within the MLs stipulated by Commission Regulation (EU) 2023/915 for oilseeds; As concentrations were likewise very low, though no specific EU ML for As in oilseeds is currently established under this regulation. Health risk modelling confirmed negligible noncarcinogenic risk across all groups and locations (maximum HI = 0.020, well below the regulatory threshold of 1.0, calculated using the updated USEPA IRIS 2025 RfD for As) and acceptable carcinogenic risk (total ILCR ≤2.81 × 10^−6^ for all groups; Cd + As only, Pb excluded per current guidance) at current consumption levels. Adult females recorded marginally higher HI and ILCR values than adult males at most sites due to their lower body weight at equivalent DI, underscoring the importance of sex‐disaggregated body weight parameters in exposure assessments. Children consistently exhibited elevated risk estimates per unit body weight relative to adults, though always within acceptable bounds, highlighting the value of including child‐specific parameters when assessing staple traditional ingredients.

Periodic heavy metal surveillance of melon seeds and other indigenous oilseeds in the Bono Region is recommended, with particular attention to seasonal variation and the potential northward spread of Hg contamination associated with ASGM activities in adjacent regions. These findings constitute a baseline dataset for national dietary exposure assessment and support the evidence base for evidence‐based food safety policy for indigenous Ghanaian oilseeds.

## Funding

No funding was received for this manuscript.

## Conflicts of Interest

The authors declare no conflicts of interest.

## Data Availability

The data that support the findings of this study are available from the corresponding author upon reasonable request.
